# Effects of Post-natal Dietary Milk Fat Globule Membrane Polar Lipid Supplementation on Motor Skills, Anxiety, and Long-Term Memory in Adulthood

**DOI:** 10.3389/fnut.2021.737731

**Published:** 2021-11-16

**Authors:** Sébastien Sultan, Jonas Hauser, Manuel Oliveira, Andreas Rytz, Nicolas Preitner, Nora Schneider

**Affiliations:** ^1^Brain Health Department, Nestlé Institute of Health Sciences, Nestlé Research, Société des Produits Nestlé S.A., Lausanne, Switzerland; ^2^Clinical Research Unit, Nestlé Research, Société des Produits Nestlé S.A., Lausanne, Switzerland

**Keywords:** neurodevelopment, nutrition, polar lipids, learning, memory

## Abstract

Early life nutrition critically impacts post-natal brain maturation and cognitive development. Post-natal dietary deficits in specific nutrients, such as lipids, minerals or vitamins are associated with brain maturation and cognitive impairments. Specifically, polar lipids (PL), such as sphingolipids and phospholipids, are important cellular membrane building blocks and are critical for brain connectivity due to their role in neurite outgrowth, synaptic formation, and myelination. In this preclinical study, we assessed the effects of a chronic supplementation with a source of PL extracted from an alpha-lactalbumin enriched whey protein containing 10% lipids from early life (post-natal day (PND) 7) to adulthood (PND 72) on adult motor skills, anxiety, and long-term memory. The motor skills were assessed using open field and rotarod test. Anxiety was assessed using elevated plus maze (EPM). Long-term object and spatial memory were assessed using novel object recognition (NOR) and Morris water maze (MWM). Our results suggest that chronic PL supplementation improved measures of spatial long-term memory accuracy and cognitive flexibility in the MWM in adulthood, with no change in general mobility, anxiety and exploratory behavior. Our results indicate memory specific functional benefits of long-term dietary PL during post-natal brain development.

## Introduction

Development of the mammalian brain is a complex process, which continues long after birth and can be influenced by both genetic and environmental factors, such as nutrition (1). Post-natal neurodevelopment is essential to support optimal brain connectivity that serves the development and maturation of important functions, including sensory processes, motor and cognitive functions. Post-natal development includes multiple processes (e.g., synaptogenesis and myelination) that require supply of nutrients, including lipids (an essential for building cellular membranes). Lipids are regulators of key cellular functions supporting brain connectivity such as synaptic transmission, cell signaling and myelination (2–4). In mammals, early life nutrition is provided by breast milk, in which lipids are the second constituent in quantity. Breast milk lipids promote general growth, maturation of the immune system and neurodevelopment (5, 6). Lipid composition of breast milk is strongly dependent on maternal nutrition as well as other environmental and genetics factors (5, 7, 8). Breast milk lipids are stored in lipid droplets surrounded by the milk fat globule membrane (MFGM), containing polar lipids (PL), cholesterol, glycolipids and proteins (9, 10). In humans, infant formula containing MFGM has shown clinical benefits on immune defense, intestine maturation, and brain development (10, 11). Moreover, beneficial effects on cognitive development have been reported in a clinical trial assessing the effect of MFGM infant formula supplementation (12). Other studies have investigated specifically the role of MFGM lipids and concluded beneficial effects on infant development, including neurodevelopment (13). Among them, PL, such as sphingolipids and phospholipids are an important component of the brain lipidome (14) and play a critical role during cognitive development (15, 16).

Preclinical studies have investigated the underlying mechanisms mediating the positive impact of MFGM and have shown significant alteration of the brain lipid composition and modulation of cognition (13). Rat pups artificially reared with formula containing MFGM showed difference in brain lipid composition and accelerated developmental reflex maturation compared to rats fed with non-MFGM control formula (17). Early life MFGM supplementation of growth-restricted rats resulted in improved cognitive performance, as well as an increase in mRNA expression of brain derived neurotrophic factor (18). Previous work reported that a milk replacement supplemented with prebiotics, bovine MFGM and lactoferrin modulated structural brain development in piglets (19). However, another piglet study reported no effect of MFGM supplementation alone on behavioral performance (20). In a rat study, maternal supplementation with a complex lipid mixture containing gangliosides and phospholipids, extracted from bovine buttermilk containing MFGM, showed transient early life differences in brain lipid content and composition with no benefit in any of the behavioral functions tested (21). In contrast, long-term post-natal supplementation with a mixture of milk lipids derived from MFGM showed improved learning performances in rats (29, 30). Together these studies demonstrate an influence of MFGM and/or its lipid components on brain development. However, the benefit on cognitive performance is still unclear for MFGM as well as for its various component (e.g., lipids). To date, there is limited knowledge on the impact of long-term post-natal dietary PL supplementation on cognitive function.

Therefore, in the present study, we assessed the long-term impact of a dietary supplementation with a source of PL, extracted from an alpha-lactalbumin enriched whey protein containing 10% lipids, on a battery of behaviors in young adult rats. The tasks selected aimed at providing a broad coverage of behavioral functions, including locomotion, motor coordination, anxiety, recognition, and spatial long-term memory. This study aims to provide a better understanding of the specific role played by the lipid fraction of MFGM in brain development.

## Methods

### Animals

Animal housing and behavioral testing were performed by Amylgen (Montferrier-sur-Lez, France). The experimental procedure involving animals was reviewed by ethics committees and conducted in strict adherence to the European Union directive of September 22, 2010 (2010/63/UE). Amylgen's authorization was delivered by the Direction Régionale de l'Alimentation, de l'Agriculture et de la Forêt du Languedoc- Roussillon (agreement #A 34-169-002; May 02, 2014).

Pregnant Wistar rats were purchased from JANVIER (Saint Berthevin, France) and were kept under a 12 /12 h light/dark cycle (lights off at 07:00 p.m.) in a temperature and humidity-controlled environment (21–22°C, 40–60%) with food and water *ad libitum*. Within 48 h after birth, litters (*n* = 5) were adjusted to eight males, based on the body weight, and fostered to receiver lactating dams. Males were selected to avoid potential effects of estrus cycle in our readouts (22).

At post-natal day (PND) 7, pups from each litter were randomly allocated to their experimental groups (*n* = 14 males per group) and supplemented with PL extract (PL animals) or with control lipid blend (control animals). The experiment was designed using a stratified randomization that balance the litter effect by allocating half of the animals (*n* = 4) of each litter to each of the two experimental groups. At weaning, two animals had to be replaced in the PL group and came form an additional litter.

At PND 21, littermate rats were housed in pairs, under a 12/12 h light/dark cycle (lights off at 07:00 p.m.) in a temperature and humidity-controlled environment (21–22°C) with food and water *ad libitum* until the end of the study. Body weight was monitored through the study and individual food consumption was estimated from cage consumption starting at weaning.

### Nutritional Intervention

The PL group received a source of PL extracted using a conventional Folch method (23); not intended for human consumption from a unique processed α-lactalbumin enriched whey protein concentrate ingredient containing 10% lipids (24). The polar lipid extraction included a step designed to remove traces of organic solvent during which samples were exposed to high vacuum in a desiccator. The PL extract (81.4 ± 6.5 g/100 g dry matter) was composed of fat 61.65 ± 1.48 g/100 g, proteins 5.72 ± 0.29 g/100 g, and ash 4.16 ± 0.26 g/100 g of extract (carbohydrates were below detection limit). The fat portion of the PL extract was composed of saturated fatty acids 36.28 ± 2.18 g/100 g, mono-unsaturated fatty acid 15.96 ± 0.96 g/100 g, poly-unsaturated fatty acids 5.93 ± 1.25 g/100 g, total trans-fatty acids 1.57 ± 0.34 g/100 g of PL extract (mean ± expanded uncertainty at the 95% confidence level and a coverage factor *k* = 2). The polar lipids of the PL extract were composed of 14.6 g of sphingomyelin, 14.5 g of phosphatidylcholine, 1.1 g of phosphatidylserine, 5.5 g of phosphatidylethanolamine and 1.1 g of phosphatidylinositol for 100 g of extract. To ensure energy balance intake, the control group received an equivalent amount of a blend of oils containing corn oil (35%), soybean oil (50%) (both from Sofinol S.A., Manno, Switzerland) and cocoa butter (15%) (Gerkens Cacao®, EJ Deventer, The Netherlands).

From PND 7 until weaning (PND 21), each rat in the PL group received 3.37 mg of PL extract, containing 1.28 mg of PL, per gram of body weight once a day. This dose was selected based on a previous study (25). The PL supplement was warmed at 52°C and emulsified in water before supplementation, while the control lipid blend was administered as such. Both PL and control lipid supplements were prepared to provide the same amount of lipid per gram of body weight. The daily dose of lipid supplement was administrated by oral gavage (10 μl per g of body weight) in addition to dams' milk, with adapted feeding tubes (product number FTP 20–30, 20 gauge; Instech Laboratories, Plymouth, PA, USA).

After weaning, rats were allowed unlimited access to water and diet. To ensure nutrient requirements among different life stages, animals received standard semi-purified diet AIN-93G diet from PND 21 to 40 followed by AIN-93M diet until the end of the study (26). The rats in the PL group received the same semi-purified diet containing 1.3% of PL extract [adapted from Haramizu et al. (27)]. Diets were composed to have the same fat content. The experimental timeline is described in Supplementary Figure 1.

### Behavioral Testing

Animals (*n* = 14 per group) were sequentially assessed in the following behavioral test battery: elevated-plus maze (EPM), open field (OF), novel object recognition (NOR), rotarod test and Morris water maze (MWM). Behavioral testing was initiated at 49 days of age (young adult). EPM was conducted at PND 49, OF at PND 50, NOR at PND 51 and 52, rotarod from PND 56 to 59 and MWM from PND 62 to 72 (Supplementary Figure 1). Animals were tested during the first 6 h of the light cycle of the light/dark cycle and were transported to the core facility 1 h before the beginning of the behavioral test. Experimenters were blind to treatment throughout the study.

### Elevated Plus Maze

Each rat was placed at the center of the EPM facing the closed arm. The EPM consisted of clear Plexiglas with two opened arms (23.5 cm × 8 cm) and two closed arms (23.5 × 8 × 20 cm high), extending from a central platform and placed 50 cm above the floor. The exploratory behavior was recorded for 10 min by tracking the gravity center of the animals with Ethovision® XT 12.0 (Noldus Information Technology, Wageningen, the Netherlands). During each trial, the number of open or closed arm entries was measured as well as the percentage of time spend in open or closed arms per visit.

### Open Field

Animals were placed in a squared open field (50 cm × 50 cm × 50 cm high) made of white Plexiglas with a floor equipped with infrared light emitting diodes (Noldus, Wageningen, the Netherlands). The exploratory behavior was recorded by an IR-sensitive camera (Basler AG) and analyzed with Ethovision® software (Noldus, Wageningen, the Netherlands) for 25 min. The locomotor activity was assessed by measuring the total distance traveled (cm) and speed (cm/s). The level of anxiety was assessed by quantifying the percentage of locomotion in the periphery of the open field.

### Novel Object Recognition

The NOR test was conducted in the same arena as OF and thus, the OF test was considered as a habituation session. During the training session on day 1, ~24 h after the OF test, animals were placed in the arena, in which two identical objects (50 ml plastic vials with caps) were placed at two opposite edges of the central area. The exploratory behavior was then recorded for 10 min by using the Ethovision nose tracking protocol to measure the locomotor activity (total distance in cm), the number (interaction frequency) and duration of contacts (interaction time) with each object.

Session 2—test session. On day 2, one of the two objects presented during the training session was replaced by a novel object (a soft plastic chair feet protection) with different color, shape, and texture. Rats were free to explore both novel and familiar objects for 10 min. The exploratory behavior was recorded by using the Ethovision nose tracking protocol as described for the training session (day 1).

The discrimination index for interaction time was computed as follows: (new object contact time – old object contact time)/(new object contact time + old object contact time). The similar index was computed for interaction frequencies. The result can vary between +1 (more time spent with the novel object) and −1 (more time spent with the familiar one) and 0 indicates a null preference.

### Rotarod Test

Motor coordination and balance were assessed using a rotarod apparatus (Harvard Apparatus, Panlab 76-0772; 6 cm in diameter). The test was done in 2 sessions. During the training session (PND 56–58), animals stayed on the rotarod apparatus during three trials per day (60 s per trial, inter-trial time 10 min, constant speed 5 rpm).

During the test session (PND 59), the animals were placed on the accelerating rod (from 4 to 40 rpm) with a cut off time at 300 s. The latency to fall was recorded during three consecutive trials (10 min inter-trial interval) to determine possible differences in motor coordination among the groups.

### Morris Water Maze

The water-maze was a circular open swimming arena (diameter 150 cm, height 40 cm) filled with opaque water at 23 ± 1°c, under dim light and in a room containing external cues (sink, contrasted posters, shelves).

#### Visible Platform Task

At PND 62, animals were pre-trained in the swimming arena using a platform equipped with a visual cue. This was done to familiarize the animals with the water maze and water exposure stress, as well as to allow evaluation of potential differences in visual or motor functions. Animals were placed in the swimming arena to find a circular escape platform made of Plexiglas (diameter 10 cm) immersed under the water surface (2 cm) marked with a visible cue. Three trials were conducted (20 min intertrial interval) to measure the escape latency (i.e., time it takes for the animals to find the platform). Animal failing to find the platform within 90 s were guided to the escape platform by the experimenter.

#### Spatial Learning and Long-Term Memory

The spatial learning was conducted during 5 consecutive days (PND 63–67). Animals were placed in the swimming arena, filled with opaque water to find a circular escape platform made of Plexiglas (diameter 10 cm) immersed under the water surface (2 cm). Three trials were conducted per day (20 min intertrial interval), during which the escape latency (i.e., time it takes for the animals to find the platform) was measured. Animals failing to find the platform within 90 s were guided to the escape platform by the experimenter. A relatively small number of trials was selected to increase the difficulty of the task. A probe test was performed 24 h after the last learning trial (PND 68). Learning was assessed by reduced latency to find the platform during the 5-day spatial learning trials. During the probe trial, the escape platform was removed, and the rats were free to explore the swimming arena for 60 s. The swimming arena was virtually divided into four equal quadrants and the percentage of time spent in each quadrant was measured. Long-term memory was assessed by percent time spent in the target quadrant during the probe trial (percentage time in quadrant), crossing of an area with the exact size (site-crossing) or twice the size of the platform (annulus crossing).

#### Reversal Learning and Long-Term Memory

Reversal learning was assessed for 3 days following the probe test (PND 69–71). The same procedure was used as during the spatial learning task, except the escape platform was relocated to the opposite quadrant of the arena. Twenty-four hours after the reversal learning (PND 72), a reversal probe trial was conducted and analyzed as previously described. Latency to reach the platform was manually recorded during visual training and acquisition trials. During all probe trials, animals' behavior was recorded using the Ethovision® software 13.0 (Noldus, Wageningen, the Netherlands).

### Statistical Analysis

For the EPM, OF, NOR and rotarod tests (training session), mean differences were tested for statistical significance by using a bilateral unpaired *t*-test with Welch's correction. Statistical differences between groups were tested with a Two-way repeated measures ANOVA for: body weight, estimated food consumption, test session of the rotarod test and learning tests of the MWM. During the probe trials, the time spent in the target quadrant was tested with a one-way ANOVA followed by uncorrected Fisher's LSD. And mean differences for the number of site-crossing and annulus crossing were tested with a bilateral unpaired t-test with Welch's correction. All statistical analyses were performed by using GraphPad Prism (version 9.0.0 for Windows GraphPad Software, San Diego, California USA). Experimenters were blind to treatment throughout the study.

## Results

To measure the impact of the supplementation with the PL extract on behavioral functions, we performed a battery of behavioral tests from PND 49–72. We assessed: anxiety in the EPM (PND 49), exploratory behavior in the OF (PND 50) followed by recognition memory in the NOR test (PND 51–52), motor function with the rotarod test (PND 56–59), and spatial and reversal learning were measured in the MWM (PND 62–72). There was no difference in body weight, estimated food intake (Supplementary Figure 2), anxiety, exploratory behavior, recognition memory and motor function between the two experimental groups throughout the study (Table 1).

**Table 1:** Behavioral tests results summary.

**Behavioral test**	**Behavior and function**	**Readout 1, values: average ± SD difference between groups**	**Readout 2, values: average ± SD difference between groups**	**Readout 3, values: average ± SD difference between groups**
**Elevated-plus maze**	Anxiety	**Time spent in closed arms (% of total time)** • PL: 59.4 ± 9.9, • Control: 56.9 ± 12.1 •Non-significant	**Number of entries** **in closed arms** • PL: 31.7 ± 10.6 • Control: 31.5 ± 9.09 •Non-significant	**Number of entries in opened** **arms** • PL: 8.2 ± 6. • Control: 8.3 ± 5.3 •Non-significant
**Open Field**	Locomotion and anxiety	**Total locomotor activity (m)** • PL: 103.3 ± 14.2 • Control: 97.8 ± 8.8 • Non-significant	**Anxiety—% of locomotion in periphery of arena** • PL: 85.1 ± 7.9 • Control: 84.4 ± 5.3 • Non-significant	
**Novel object recognition**. (Same object session)	Exploratory behavior	**Total locomotor activity (m)** • PL: 28.4 ± 5.9 • Control: 32.8 ± 7.2 • Non-significant	**Object 1—interaction frequency (%)** • PL: 46.1 ± 14.03 • Control: 50.9 ± 13.4 • Non-significant	**Object 1—interaction** **time (%)** • PL: 51.1 ± 9.3 • Control: 53.9 ± 12.6 • Non-significant
**Novel object recognition**. (Novel object session)	Long term recognition memory	**Total locomotor activity (m)** • PL: 31.6 ± 5.4 • Control: 31.2 ± 4.3 • Non-significant	**Novel object interaction frequency (%)** • PL: 60.2 ± 12.5 • Control: 62.4 ± 13.3 • Non-significant **Discrimination index** • PL: 0.20 ± 0.25 • Control: 0.24 ± 0.26 • Non-significant	**Novel object interaction** **time (%)** • PL: 61.09 ± 13.04 • Control: 63.9 ± 14.1 • Non-significant **Discrimination index** • PL: 0.22 ± 0.26 • Control: 0.27 ± 0.28 • Non-significant
**Rotarod training session**. Constant speed	Motor learning	**Latency to fall (sec) day 1** • PL: 40.9 ± 15 • Control: 41.5 ± 15.2 • Non-significant	**Latency to fall (sec) day 2** • PL: 52.2 ± 9.2 • Control: 58.8 ± 2.9 • Significant: unpaired *t*-test with Welch's correction, *P* = 0.02	**Latency to fall (sec)** **day 3** • PL: 58.7 ± 3.5 • Control: 58.6 ± 2.6 • Non-significant
**Rotarod testing session**. Accelerating speed 4–40 rpm	Motor coordination	**Difference in active rotation Latency to fall (sec)—last vs. first trial-**(3 trials per group) • Non-significant • Two-way repeated measures ANOVA Interaction Groups x Trial *F*_(2;52)_ = 2.1, *p* = 0.13		

*Experimental groups (n = 14 males per group) PL: animals supplemented with polar lipids. Control: animals supplemented with the control lipid blend. All data are expressed as mean ± SD. Statistical significance is indicated in each cell*.

**Figure 1:**
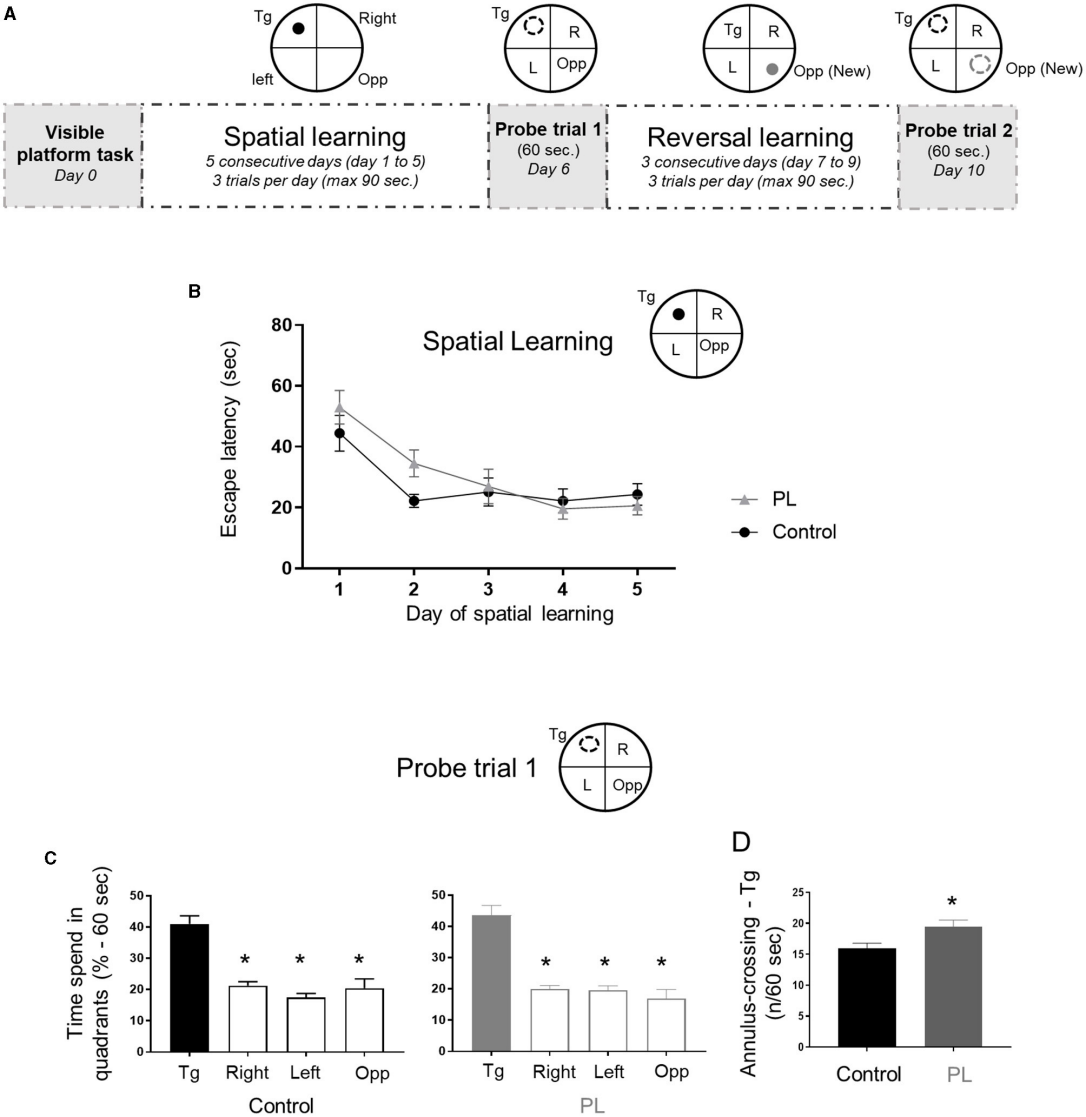
Spatial learning and target long-term memory. **(A)** Experimental timeline, 2 experimental groups (*n* = 14 males) were tested in the Morris water maze test to assess spatial learning and reversal learning performances. Animals supplemented with polar lipids (PL) showed similar learning performances than control animals during **(B)** the initial spatial learning as shown by the decreased time to find the escape platform. **(C)** During the probe trial 1 both groups spend significantly more time in the target quadrant compared to the other three quadrants (Tg, * = *P* < 0.05 for each quadrant compared to quadrant Tg). **(D)** During the probe trial 1, PL animals showed a higher number of annulus crossing than control animals (* = *P* < 0.05). All data are expressed as mean ± SEM.

We used the MWM to test animals' spatial reference long-term memory. After a visible platform task showing no difference between the two experimental groups (time to find the platform, average ± SD: PL = 31.3 ± 13.2 s vs. Control = 28.1 ± 11.3 s; unpaired *t*-test with Welch's correction, *P* > 0.05), spatial learning was measured during 5 consecutive days (Figure 1A). Both experimental groups improved their spatial learning during acquisition, but there was no difference between the groups (Figure 1B, Two-way repeated measures ANOVA, interaction time x groups *F*_(4, 52)_ =1.5, *P* = 0.2). Similar number of rats required guidance to the platform after the time limit across the 5 days of acquisition, suggesting that this procedure did not differentially impact learning performance in either experimental groups (data not shown). The probe trial was conducted 24 h after the learning session by determining the time spent in the target quadrant and the number of site and annulus crossing to assess the animals' ability to remember the spatial location of the platform. Both groups exhibited a significant preference for the target quadrant (Figure 1C; One-way ANOVA followed by uncorrected Fisher's LSD test to compare the percentage of time in the Tg quadrant to the other quadrants, PL = *F*_(3, 52)_ = 28.9, *P* < 0.001 and Control = *F*_(3, 52)_ = 24.1, *P* < 0.001). When the spatial long-term memory accuracy was assessed by determining the number of site (1 × platform diameter) and annulus (2 × platform diameter) crossings, PL animals exhibited more focal search around the escape platform area as shown by a significantly higher annulus crossing than control animals (Figure 1D, unpaired *t*-test with Welch's correction, P = 0.018). No significant differences were found for the number of site-crossing (average ± SD, PL = 8 ± 1.9 vs. Control = 7.4 ± 2.1; unpaired *t*-test with Welch's correction, *P* > 0.05).

**Figure 2:**
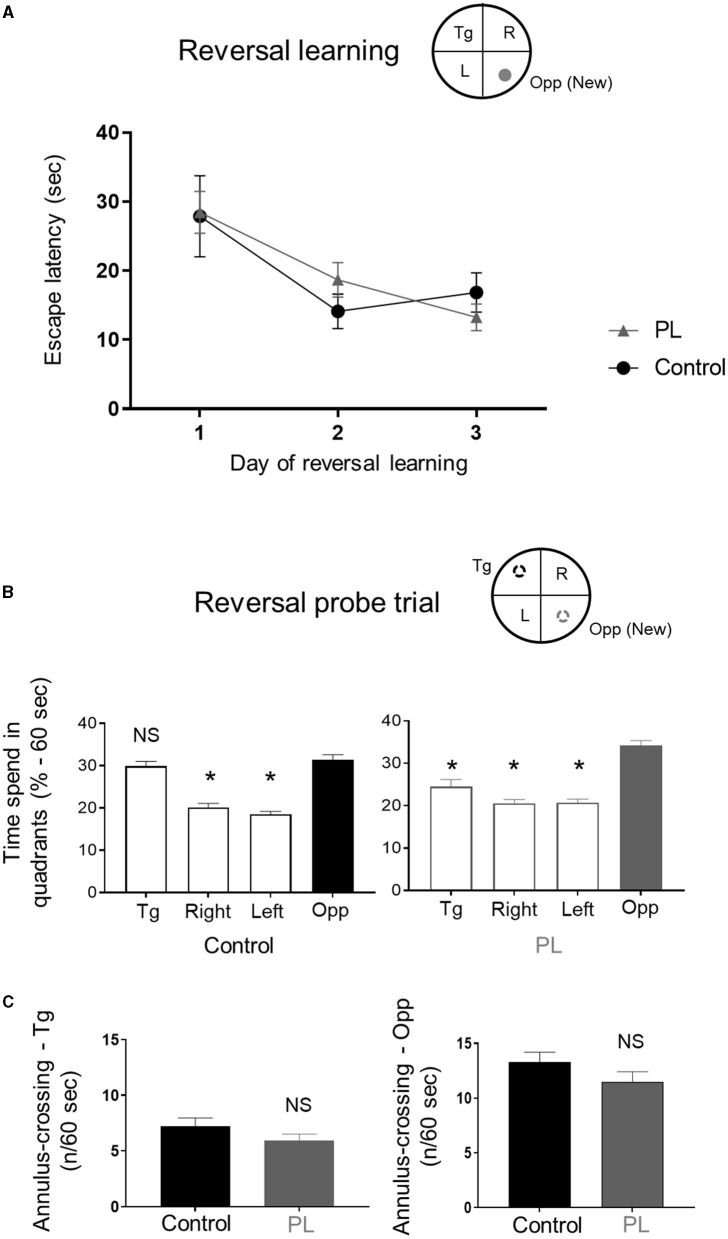
Reversal long-term memory. Animals supplemented with polar lipids (PL) showed similar learning performances than control animals during **(A)** the reversal learning as shown by the decreased time to find the new escape platform. **(B)** During the reversal probe trial, the time spend by PL animals in the new target quadrant (Opp) was significantly higher than the other quadrants (left panel) while control animals spend a similar amount of time in both the new (Opp) and old (Tg) target quadrants (right panel, * = *P* < 0.05 for each quadrant compared to Opp quadrant). **(C)** Both groups showed a similar number of annulus crossing for both the new (Opp) and initial (Tg) platforms. All data are expressed as mean ± SEM.

We then performed a reversal spatial learning in the MWM followed by a reversal probe trial. Both experimental groups quickly learned the new position of the platform as shown by the decreased escape latency from day 1 to 3. There was no significant difference between the groups (Figure 2A, Two-way repeated measures ANOVA, time x groups *F*_(2, 26_) =1.005, *P* > 0.05).

During the reversal probe trial, control animals exhibited a preference for both the reversal (Opp) and the spatial learning (Tg) quadrants (Figure 2B, left panel; One-way ANOVA followed by uncorrected Fisher's LSD to compare the percentage of time in the Opp quadrant to the other quadrants, *F*_(3, 52)_ = 43.01, *P* < 0.001). The PL animals spent more time in the reversal quadrant compared to all other quadrants (Figure 2B, right panel; One-way ANOVA followed by uncorrected Fisher's LSD test to compare the percentage of time in the Opp quadrant to the other quadrants, *F*_(3, 52)_ = 29.5, *P* < 0.001). There was no significant difference between the two groups for both the number of site-crossings (average ± SD, Opp: PL = 5.5 ± 2.2 vs. Control = 6.6 ± 2.3; Tg: PL = 3.2 ± 1.6 vs. Control = 4.4 ± 2.1; unpaired *t*-test with Welch's correction, *P* > 0.05) and annulus crossing in the original and new target quadrants (Figure 2C, left panel (Tg) and right panel (Opp); unpaired *t*-test with Welch's correction, *P* > 0.05).

## Discussion

We investigated the impact of long-term post-natal PL supplementation, extracted from an alpha lactalbumin-enriched whey protein concentrate, on a battery of behavioral tests in young adult rats. Our results showed an improvement in measures of spatial long-term memory accuracy and cognitive flexibility in the MWM with no effect in locomotion, motor coordination, anxiety, or object recognition memory in PL animals.

The post-natal period is critical for brain development. After weaning, the rat brain is not fully mature and some brain areas, such as the hippocampus, continue to grow until PDN 90 (28). In our study, PL supplementation started at PND 7 and behavioral testing was initiated at PND 49, while animals were still supplemented. In the MWM test, both groups showed similar performance during the acquisition and the probe trial, suggesting that the PL supplementation did not affect spatial learning or long-term memory. Noteworthy, as we recorded manually the latency to reach the platform, we could not exclude potential differences in swim speed. However, based on the absence of change in any of the locomotor or coordination assessments (distance moved in the open field, distance moved in the EPM, rotarod latency to fall), it is unlikely that the observed effects of PL supplementation were purely due to differences in swim speed. One additional point worth further discussion is the size of the water maze used in our study; for feasibility reasons we used a 150 cm diameter water maze, which is smaller than the more common 200 cm diameter used in rat studies. This difference might have resulted in an easier task for the rats, thus potentially reducing the opportunity to detect differences between our experimental groups. Therefore, to investigate more subtle effects on spatial long-term memory, such as differences in search strategy, we used two additional indices of spatial reference long-term memory, the numbers of site and annulus crossing. No significant difference was noted between the groups in the number of site crossing. However, PL animals showed an increased number of annulus crossing, suggesting that the animals used a more focused search strategy in the probe trial, potentially indicating a more precise memory trace for the platform location. In previous animal studies, rats exposed to long-term post-natal supplementation (from PND 10 to PND 70 or 80) with a complex milk lipid concentrate enriched in ganglioside and phospholipids showed some improvements during the acquisition of spatial memory in the MWM. Specifically, supplemented rats exhibited a shorter latency to enter the target quadrant during the probe trial (29, 30). While the experimental treatment between these studies and ours are different, all three studies contained PL and resulted in some improvements in spatial memory. Similar to our study, the PL supplement had no significant effect on anxiety, assessed in the dark light boxes and the elevated plus maze (30), suggesting that the benefits observed in spatial memory is not related to any change in anxiety. Other studies used different treatment windows and/or models. For example, mice supplemented from PND 16 to 44 with a diet enriched in large lipid droplets containing milk phospholipid showed better scores in T-maze alternation and NOR performance when tested between PND 35 and 37. Interestingly, while the discrimination index measured with the NOR was still improved into adulthood (PND 78) no difference was found in T-maze spontaneous alternation (31). Conversely, no benefit of our supplementation was noted during the NOR test, suggesting the behavioral benefits might depend on the treatment windows and experimental paradigms and designs. In a clinical study investigating the impact of MFGM on cognition, Timby and colleagues reported a similar transient positive effect on cognition visible at 2 years of age but not visible at a 6.5-years follow-up visit (12, 32). Thus, supporting the hypothesis that potential benefits of MFGM might be transient depending on the treatment windows or that the long-term effects are subtle and difficult to detect.

The reversal training was also conducted to assess the animals' ability to learn the new position of the escape platform and to extinguish the memory for the previous platform location (33). No significant difference was noticed during the reversal learning session between groups, indicating that both groups were able to learn the new position of the target with similar performance. During the reversal probe trial, PL animals showed a preference for the new target quadrant, while control animals showed a preference for both the new and the original target quadrants. This result could be interpreted as an improvement in behavioral flexibility in the PL animals.

All together these results suggest some improvement in spatial long-term memory and behavioral flexibility in animal supplemented with the PL extract. This behavioral improvement might be supported by a more efficient information processing in the hippocampus and/or prefrontal cortex. Indeed, these regions play key roles in spatial memory and behavioral flexibility (34–36). In a recent piglet study, an early life supplantation with a source of PL has shown a small increase in hippocampal maturation (37). Therefore, it is conceivable that the PL extract used in our study may have promoted maturation of key brain structures involved in spatial memory functions, such as hippocampus, due to the key role it plays as a building block for cellular membrane formation or myelination processes necessary for neuronal network development. Furthermore, phospholipids and their metabolites are known to be essential for neurotransmission and synaptic plasticity, both of which are essential for memory formation (38). Thus, in addition to its role in brain structural maturation, PL supplementation may have promoted functional maturation at a cellular level that supports neuronal network activity and plasticity necessary for optimal brain functions, which may underlie the observed long-term memory improvement. Moreover, new neurons are continually generated in the hippocampus through the lifespan from adult neural stem cells (aNSC). This process called adult neurogenesis has been shown to play a critical role in hippocampal plasticity supporting hippocampal-dependent processes such as reversal learning and spatial learning and memory (39, 40). Lipids metabolism has been shown to influence aNSCs proliferation and neuronal differentiation (41). In adult rats, supplementation with one polar lipid, phosphatidylserine, has been associated with increased aNSCs proliferation (42). Therefore, it is conceivable that the PL supplementation could have supported adult neurogenesis, not only by providing building blocks used to synthesize the new neurons but also by supporting aNSCs metabolism.

To increase the translational value of our study we decided to use dietary supplementation in a healthy model. PL extract was supplemented in addition to the maternal milk before weaning and regular nutrition after weaning in healthy rats. Some previous studies, in contrast, have used a growth-restricted rat model to examine the benefits of early life MFGM supplementation on brain development and behavioral function late in life (18, 43). In these studies, the growth restricted model was used to simulate nutritional deficits during early life. Post-natal MFGM supplementation (from PND 2–21) was able to compensate for brain and cognitive deficits induced by growth restriction during the post-natal period and into adulthood. Nevertheless, no benefit of MFGM supplementation on behavior was reported in the control non-growth-restricted animals (18). In another study, acceleration of some developmental reflexes has been observed in rats artificially reared with a milk replacer supplemented with MFGM, suggesting a beneficial effect of MFGM during post-natal neurodevelopment (17). To note, artificial rearing methods have been shown to be associated with negative impact on the maturation of physiological functions, including anxiety-like behavior (44) and changes in brain metabolism (45). This suggests that the observed benefits following MFGM supplementation in artificially reared rodents could be specific to the experimental model. On the contrary, in young pigs, artificial rearing has been shown not to elicit detrimental effects on brain development and behavior (46). In a study assessing the effect of a supplementation with phospholipids and gangliosides during the neonatal period, better performance in a spatial T-Maze task was observed in supplemented piglets (47). In another piglet study, animals artificially reared with a milk replacer supplemented with a blend containing prebiotics, MFGM and lactoferrin from 2 to 31 days of age, were reported to have an increased brain maturation concomitant with subtle changes of T-maze performance (19). While an additional piglet study, reported no change in recognition memory performance, assessed with a NOR test in response to supplementation of milk replacer with MFGM only (20). The aforementioned studies reporting effects of supplementation with various sources of PL seem to utilize models known to elicit some degree of neurodevelopmental deficits. Interestingly, in the studies using healthy models, the effects observed were often subtle and more related to an accelerated brain maturation. Extrapolating these findings to a neurodevelopmental disorder situation, one could hypothesize that a similar early life PL supplementation could mitigate the associated brain development delays.

In conclusion, we have shown, in a healthy preclinical model, that post-natal PL supplementation exerted some positive impact on long-term spatial memory accuracy and cognitive flexibility in adulthood. No effect of the supplementation, however, was found on locomotion, motor coordination, anxiety, or recognition memory in adulthood. As the effect observed on spatial learning was subtle, further exploration on the impact of our PL extract on memory would be needed to reach a firm conclusion. Nevertheless, these findings add to previously reported effects in models of brain developmental delays, which suggest that nutritional building blocks such as PL are critical dietary components during the plastic phase of brain development that may influence the cognitive performance.

## Data Availability Statement

The raw data supporting the conclusions of this article will be made available by the authors, without undue reservation.

## Ethics Statement

The animal study was reviewed and approved by Direction Régionale de l'Alimentation, de l'Agriculture et de la Forêt du Languedoc- Roussillon - France -(agreement #A 34-169-002; May 02, 2014).

## Author Contributions

SS, MO, JH, and NS contributed to the study design. SS, JH, and AR collected and analyzed the data. All authors contributed to the article and approved the submitted version.

## Funding

The authors declare that this study received funding from Société des Produits Nestlé S.A. The funder had the following involvement with the study: study design, data analysis, decision to publish, and preparation of the manuscript.

## Conflict of Interest

SS, JH, MO, AR, NP, and NS are employees of Société des Produits Nestlé S.A.

## Publisher's Note

All claims expressed in this article are solely those of the authors and do not necessarily represent those of their affiliated organizations, or those of the publisher, the editors and the reviewers. Any product that may be evaluated in this article, or claim that may be made by its manufacturer, is not guaranteed or endorsed by the publisher.
