# Supplementary figures and images for: Effects of Post-natal Dietary Milk Fat Globule Membrane Polar Lipid Supplementation on Motor Skills, Anxiety, and Long-Term Memory in Adulthood
Source: Front Nutr. 2021 Nov 16;8:737731. doi: 10.3389/fnut.2021.737731 (PMC8637295; doi:10.3389/fnut.2021.737731)

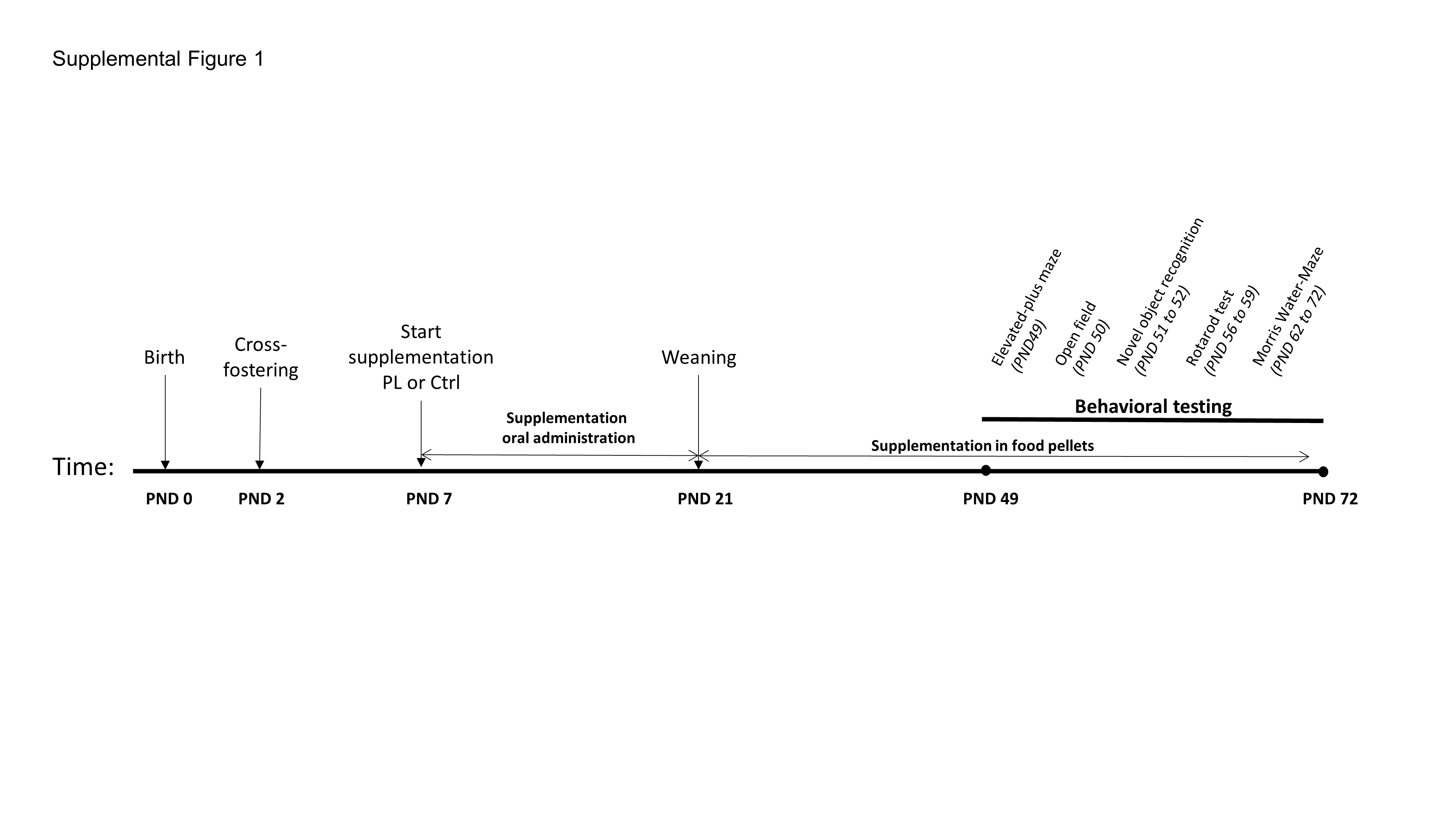

Supplement: Supplementary file 1 [file Image_1.tif]

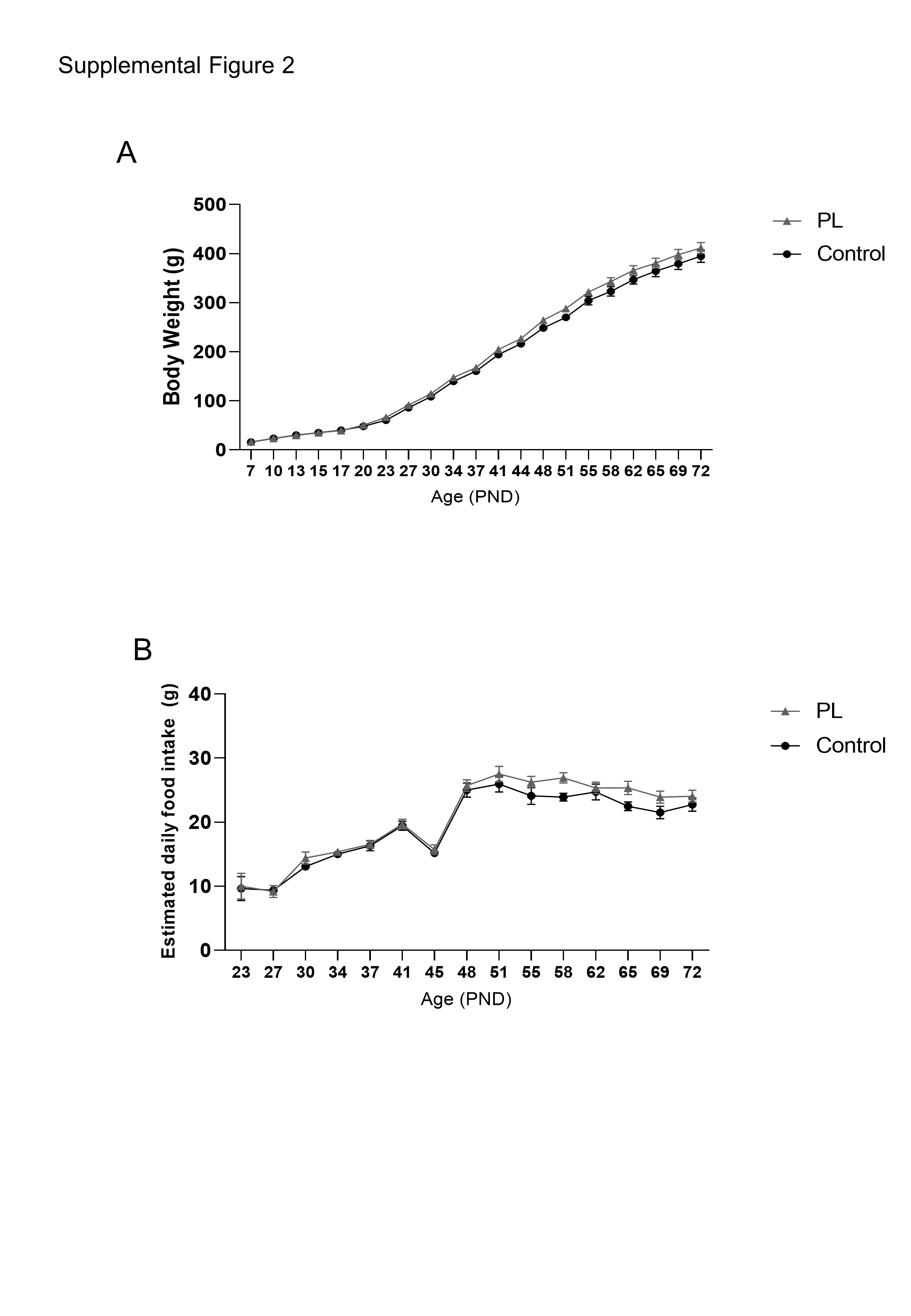

Supplement: Supplementary file 2 [file Image_2.tif]
